# Effects of a kennel conditioning protocol on transport-induced stress in minipigs: insights from heart rate variability and salivary cortisol

**DOI:** 10.1007/s11259-026-11259-4

**Published:** 2026-07-10

**Authors:** Emma Tognetti, Aurora Paganelli, Martina Felici, Micaela Sgorbini, Antonio Lanatà, Paolo Baragli, Fabio Anastasio Recchia, Angelo Gazzano

**Affiliations:** 1 Department of Veterinary Sciences, University of Pisa, Viale delle Piagge 2, Pisa, 56122 Italy; 2 Institute of Life Sciences, Scuola Superiore Sant’Anna, Piazza Martiri della Libertà 33, Pisa, 56127 Italy; 3 Department of Agricultural and Food Science, University of Bologna, Viale Giuseppe Fanin 46, Bologna, 40127 Italy; 4 Department of Information Engineering, University of Florence, Via di S. Marta 3, Florence, 50139 Italy

**Keywords:** Miniature pigs, Transport, Stress, Training, Sample entropy, Cortisol, Welfare

## Abstract

Transportation can be a significant stressor to laboratory animals. This study evaluated the efficacy of a five-week kennel conditioning protocol in mitigating stress induced by transportation in adult minipigs. Thirteen animals were randomly assigned to the conditioned (CND) or control (CTR) group. Only the CND group underwent conditioning in the kennel that would be used for transport. After the conditioning period, both groups were subjected to simulated transport. Stress responses were assessed through salivary cortisol levels at baseline, during kennel permanence, and during simulated transport, while heart rate variability (HRV) was continuously monitored. There were no significant differences observed in salivary cortisol levels between groups. Overall, neither group showed clear physiological evidence of transport-related stress. At baseline, CTR minipigs displayed higher Sample Entropy (SampEn), a nonlinear parameter of autonomic complexity, than CND minipigs. This suggests positive emotional arousal due to training. During transport, CND minipigs exhibited a significant intragroup increase in SampEn compared to baseline, indicating differences in autonomic regulation. These results suggest that repeated handling and exposure to non-aversive experimental procedures may contribute to stress resilience, potentially reducing the detectable impact of specific transport-conditioning protocols. Our results underscore the complexity of interpreting physiological stress indicators and support the use of multimodal approaches combining complementary welfare measures. Further studies are necessary to determine the relative contributions of targeted conditioning and general husbandry practices in shaping transport responses in biomedical minipigs.

## Introduction

Transportation is a recognized stressor for laboratory and farm animals of all species. It can trigger physiological dysregulation, which may compromise animal welfare and the validity of experimental outcomes (Broom and Johnson 1993; Moberg and Mench 2000). Stress responses typically involve the activation of the autonomic nervous system and the hypothalamic-pituitary-adrenal (HPA) axis. This results in an increase in the secretion of glucocorticoids, particularly cortisol, as well as changes in autonomic balance (Sapolsky et al. 2000; von Borell 2001).

Stress evaluation in animals is typically conducted by measuring behavioral, endocrine and autonomic parameters. Salivary cortisol is a non-invasive and widely used biomarker of HPA axis activation (Mormède et al. 2007), while heart rate variability (HRV) provides a dynamic measure of cardiac autonomic regulation (Berntson et al. 1997; von Borell et al. 2007). The HRV can be analyzed in three different domains, namely time, frequency and nonlinear. Time and frequency HRV parameters, such as MeanRR (the mean of the RR intervals, in milliseconds), RMSSD (Root Mean Square of Successive Differences, in milliseconds), SDNN (Standard Deviation of NN intervals, ms), LF (low-frequency band), HF (high-frequency band), and the LF/HF ratio (low-to-high frequency ratio) are commonly used to assess sympathovagal balance in pigs and other species (von Borell and Humik 2007). Specifically, an increase in the values of certain parasympathetic features, such as MeanRR, RMSSD, and HF, indicates a shift in the autonomic nervous system toward greater parasympathetic activity. Conversely, increased values of SDNN, LF, and LF/HF suggest possible higher sympathetic activity (Shaffer and Ginsberg 2017). Moreover, nonlinear parameters such as SD1 (Standard Deviation 1), SD2 (Standard Deviation 2) and SampEn (Sample Entropy) have been investigated in pigs and horses (Byrd et al. 2020; Felici et al. 2023). SD1 correlates with HF and is identical to the time domain RMSSD. Conversely, SD2 correlates with LF (Shaffer and Ginsberg 2017). SampEn measures the regularity and complexity of a time series. A higher SampEn value indicates a more complex time series, which suggests a healthier or more adaptable physiological system. Conversely, a lower SampEn value indicates a less complex time series, which suggests a less healthy or less adaptable physiological system (Shaffer and Ginsberg 2017).

Minipigs are gaining increasing relevance in preclinical research due to their anatomical, physiological, and metabolic similarities to humans (Swindle et al. 2012). As their use becomes more widespread, refinement strategies, including those aimed at improving transport conditions, are crucial for promoting welfare and ensuring reliable experimental data (von Borell 2001; Kilkenny et al. 2010; Prescott and Lidster 2017). In this regard, the application of conditioning protocols during different phases of transport and kennel confinement has been proposed as a potential strategy to mitigate stress-induced physiological responses (von Borell and Hurnik 2007).

This study aimed to evaluate whether a kennel conditioning protocol could reduce transport-induced stress in minipigs. We hypothesized that pigs conditioned to the kennel would have lower cortisol levels and reduced sympathetic activation, as measured by HRV. These results would suggest that kennel conditioning prior to transportation improves the welfare state of minipigs.

## Materials and methods

The research protocol was approved by the Ethical and Animal Welfare Committee of the University of Pisa (n°8/24).

A total of 13 mixed-breed minipigs (Juliana minipigs × Vietnamese pot-bellied pigs), sourced from an internal breeding facility, were enrolled in this study. All the animals were 2 years old, 8/13 were female, and 5/13 were male. Females weighed 22–60 kg (median weight 40 kg), and males 21–57 kg (median weight 25 kg). All the minipigs were housed in boxes measuring 365 cm by 446 cm. The animals were group-housed according to sex, with 8 females kept in one box and 5 males in another. A separate resting area with a solid floor, straw bedding and separated from the feeding and exercise area was also provided. Straw also served as an environmental enrichment source. Animals were fed 420 g/day of specific porcine pellets (a mix of M2 pellets and Stalla Fibra pellets, Progeo, Italy) twice daily (8:00 AM and 4:00 PM) and had free access to water through pig-specific drinking nipples. Before this study, minipigs only interacted with the housing staff and the veterinarian during routine checks and no specific conditioning sessions in the presence of humans were performed. All experimental tests were carried out between 9 a.m. and 12 p.m.

### Conditioning protocol

The kennel conditioning protocol was implemented with two distinct groups, the conditioned group (CND) and the control group (CTR). Animals were first assigned identification numbers within each sex group and were then allocated to the two groups using an online random number generator, ensuring a random group composition. The CND group consisted of 4 females and 2 males, while the CTR group included 4 females and 3 males. The CND group underwent all the training sessions from week 1 to 5. The CTR group only undertook the training of weeks 1 to 3, and it was never exposed to the kennel before the data collection. During weeks 4 and 5, the CTR group continued daily training sessions with the belt-shaped electrocardiograph for 15 min per animal, to maintain familiarity with the device and avoid loss of the previously acquired habituation. The conditioning protocol is reported in Table 1 and lasted 5 weeks. At the end of the fifth week, the data collection was conducted.

**Table 1:** Conditioning protocol

Week	Dedicated time	Activity	Target
1	30 min/day/box	Socialization with the operator	The animal is comfortable with the presence of the operator
2	15 min/day/animal	Training/test area exploration	The animal is confident with the box, together with the operator
3	15 min/day/animal	Smart textile conditioning	The animal is comfortable wearing the smart textile
4	15 min/day/animal	Kennel exploration	The animal is confident with the box and the kennel. The kennel door was left open
5	15 min/day/animal	Kennel conditioning	The animal is comfortable for 15 min when closed inside the kennel
6	15 min/day/animal	Extra sessions of kennel conditioning	The animal is comfortable for 15 min when closed inside the kennel

#### Week 1 – socialization with the operator

All the animals underwent a period of conditioning in order to socialize with the operator (ET), who was in charge to conduct the conditioning and the test. The operator was a veterinarian with experience in animal training. In the early stages, the operator spent 30 min a day for 1 week near the minipigs in their housing box without direct interaction and maintaining slow movements and a calm voice so that the minipigs could acclimate to human presence. As minipigs began to exhibit curiosity rather than avoidance, the operator gradually approached, allowing the animals to observe and sniff. Sudden movements were avoided, and direct eye contact was minimized to prevent perceived threats (Forkman et al. 2007). The operator consistently associated her presence with positive reinforcement, such as feed distribution and gentle stroking. The positive reinforcement consisted of approximately 100 g of the same pelleted diet provided during routine feeding. Socialization periods were integrated into daily routines to reinforce positive associations with human presence.

#### Week 2 – training/test area exploration

For the smart textile and kennel conditioning, a single box (365 cm by 446 cm) constructed similarly to the housing box was designated for these procedures. This box had never been used to house other animals, and the minipigs were brought into it exclusively for training sessions and subsequent experimental tests. The minipigs were led one at a time into the box. In the initial exposure phase, the box door was left open, allowing the animal to voluntarily approach and explore the new space without coercion. To encourage entry, food rewards were strategically placed near the entrance and progressively moved further inside if hesitation was observed. The same type of positive reinforcement was consistently used throughout the training sessions. During this phase, the pig was allowed to move freely. Following this initial exploration, the minipig was allowed to remain inside the box for 15 min. During this time, positive reinforcement was provided in the form of the previously used food or gentle handling to create an association between the box and positive experiences. As the pig demonstrated relaxed behavior (e.g., entered the box without hesitation and explored the training/test area), the next phase of the training involved the closure of the box door.

#### Week 3 – smart textile conditioning

After becoming familiar with the training area, the animals were brought inside individually to be conditioned to wear belt-shaped electrodes (Smartex ECG, Smartex S.r.l., Pisa, Italy), which were used to record their electrocardiograms (ECG). The system consisted of smart textile electrodes that could be applied for an extended period because the material ensured reliable contact between the electrodes and the animals’ skin (Turini et al. 2022).

**Fig. 1:**
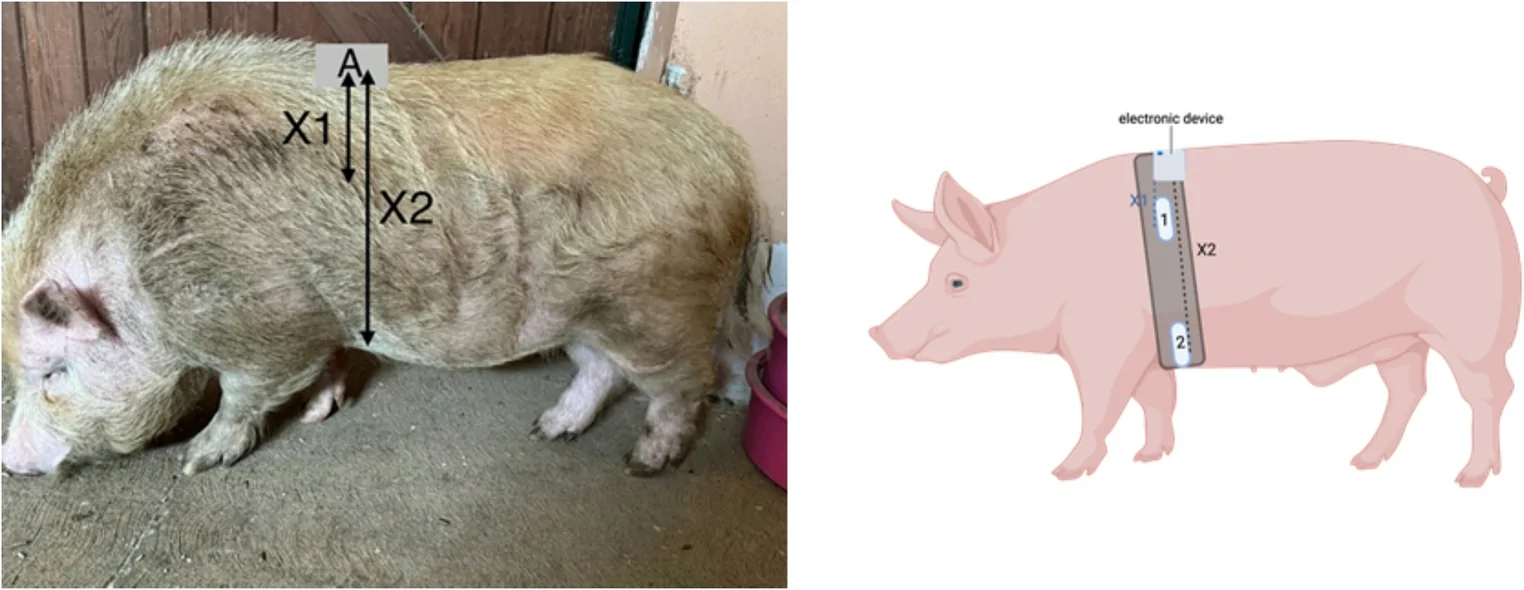
Positioning of the belt-shaped smart textile electrodes. In the figure, “A” represents the electronic device that connects via Bluetooth to the Smartex App to visualize the ECG tracings. “X1” represents the distance from the electronic device to the first electrode, and “X2” represents the distance from the electronic device to the second electrode. Each minipig was conditioned to wear the belt for 15 min every day for one week

The conditioning was carried out to ensure proper device usage without inducing stress, which could compromise data accuracy. Initially, the belt was introduced into the minipigs’ enclosure without direct application, allowing voluntary exploration. To encourage positive associations, food rewards were placed near the belt. Subsequently, a simulated application phase was conducted, where the belt was loosely wrapped around the animal’s body for short periods without securing it. The duration of exposure was progressively increased while monitoring behavioral responses. If signs of distress were observed, such as escape attempts, freezing, or high-pitched squealing, the procedure was adjusted by returning to the previous step and shortening exposure times. Once minipigs exhibited relaxed behavior during simulated applications, the belt was securely fastened in the correct position, as shown in Fig. 1.

#### Weeks 4 and 5 – kennel exploration and conditioning

The CND group underwent a structured acclimatization process designed to reduce stress and facilitate a positive association with the kennel.

The protocol was designed to gradually familiarize the minipigs with the kennel, using positive reinforcement strategies to promote voluntary engagement and minimize stress. Initially, a kennel (107 × 72 × 66 cm, D.M.O Pet Care Srl, Padua, Italy) was introduced into the minipigs’ environment without forcing interaction, allowing them to explore it. To encourage positive associations, food rewards were placed near the entrance, gradually moving further inside overtime. This step aimed to establish the kennel as a neutral or positive space rather than a confinement area.

Once pigs demonstrated voluntary approach behaviors, the next phase involved short, supervised exposures inside the kennel. The animals were gently guided inside without physical coercion, and the door was left open to prevent a sense of entrapment. During these initial sessions, food reinforcements were provided within the kennel to strengthen the association between entry and positive outcomes. As the pigs became increasingly comfortable, the door was closed for brief intervals while continuously monitoring their behavioral responses. If signs of stress, such as escape attempts, excessive vocalization, or agitation, were observed, the duration of enclosure was reduced, and additional positive reinforcement was introduced. Gradual increases in the duration of door closure were implemented as the pigs adapted, ensuring that prolonged confinement was not perceived as aversive. The task was completed when the minipig was comfortable for 15 min, closed inside the kennel.

If the CND group could not reach the target within the 2 weeks of training with the kennel, 1 extra week of conditioning sessions was conducted for animals that required them. Furthermore, if conditioning was not achieved, the animal was excluded from the experiment.

### Experimental phase: data collection for CND and CTR groups

**Fig. 2:**
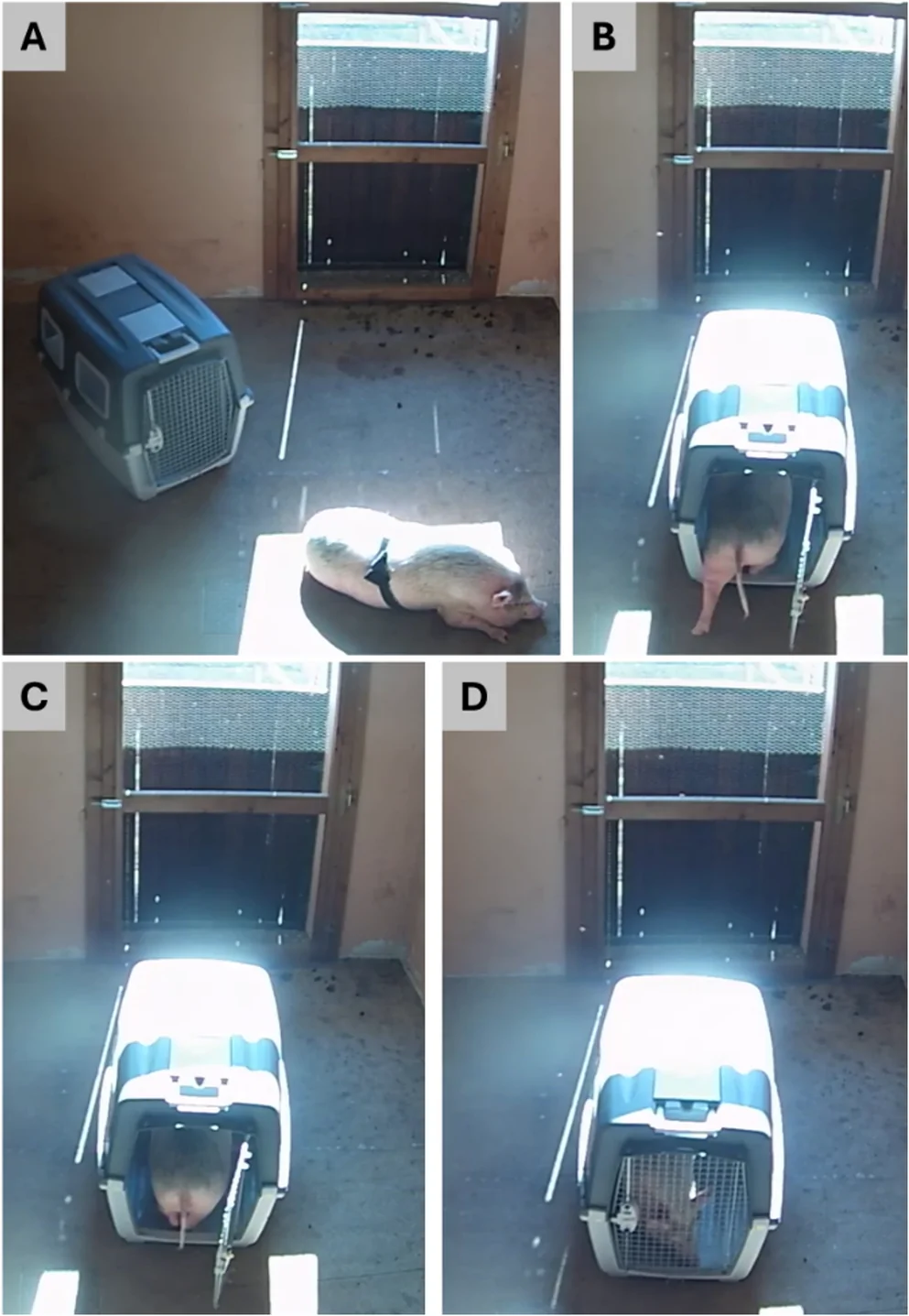
Phases 5 (**A**) and 7 (**B**-**D**) of data collection. The minipig in the photos belonged to the CND group and entered the kennel spontaneously (**B**, **C**). Photo **D** shows the kennel closed in preparation for the upcoming transport

In the experimental phase, data were collected as reported in Table 2. Phases 5 and 7 of data collection are shown in Fig. 2. The animals were not fasted before the experimental phase, and their management routine remained the same before and after.

**Table 2:** Data collection procedures

Phase	Description
1	The minipig was isolated in the test area along with the operator.
2	The operator applied the smart textile electrodes using conductive gel, and the ECG recording started. Subsequently, the first saliva sample was collected (T0).
3	If the minipig belonged to the CND group, the operator opened the kennel and waited until the minipig voluntarily entered. If it was part of the CTR group, it was helped into the kennel by the operator.
4	The minipig remained in the kennel for 20 minutes. Then, the second saliva sample was collected (T1). After the saliva sample was collected, the minipig was released from the kennel into the test area.
5	The animal was then allowed to move freely for 60 minutes within the test area.
6	Before initiating the transport simulation, a third saliva sample was collected (T2).
7	If the minipig belonged to the CND group, the operator opened the kennel door and waited for the minipig to enter spontaneously within 30 s. If the minipig belonged to the CTR group, the operator helped it into the kennel. Once inside, the kennel was loaded into the transport vehicle and followed a standardized route within the Veterinary Teaching Hospital “Mario Modenato” (San Piero a Grado, Pisa, Italy). The minipig remained inside the kennel for the entire 20-minute transport period before returning to the site of origin.
8	At the end of the transport, the last saliva sample was collected (T3).
9	Finally, the minipig was returned to its housing box.

During the whole data collection, the minipigs were equipped with the belt-shaped electrocardiograph to record the ECGs and videos were recorded to contextualize the variations in the ECG traces with what was happening during the data collection.

Salivary samples were collected during Phase 2-4-6-8, at the same time of the day (between 9 a.m. and 12 p.m.) for all animals, as shown in Table 2, using sterile cotton swabs (Salivette^®^, Sarstedt, Numbrecht-Rommelsdorf, Germany) as previously described in other species (Bonelli et al. 2019; Ogi et al. 2020). Samples were collected without physical restraint. The swab was grasped with a surgical clamp and gently introduced into the mouth through the angle of the lips while the minipigs voluntarily interacted with it. Then, the swab was placed onto the tongue for approximately 1 min. After the collection, the swab was returned into a polypropylene tube and immediately centrifuged for 10 min at 1000 rpm at 5 °C. At least 1 ml of saliva was recovered from each sample, and the samples were stored at − 20 °C until analysis. All procedures were performed by the same operator who conducted the training sessions and the experimental tests.

### Data processing

The data from the ECG monitoring system (Smartex, Smartex S.r.l, Pisa, Italy) were imported into Kubios^®^ HRV Standard (Kubios HRV software (version 2.2), Biomedical Signal Analysis Group, Department of Applied Physics, University of Kuopio, Finland) as complete ECG waveforms, and R-peaks identification on the ECGs was visually inspected and manually corrected by one of the authors (MF). MF was a veterinarian with a PhD in veterinary science. Her PhD work involved using the HRV parameter and analyzing it with specialized software. After importation and correction, five time windows of five minutes each were extrapolated from the ECG as reported in Table 3. From the five windows, time domain (i.e., MeanRR, SDNN, RMSSD), frequency domain (i.e., LF, HF, LF/HF), and nonlinear parameters (i.e., SD1, SD2, SampEn) of HRV were extrapolated. The low-frequency and high-frequency bands were set at 0.01–0.07 Hz and 0.07–1.0 Hz (Kuwahara et al. 1999), as reported in the literature for minipigs. In addition to HRV parameters, the mean heart rate (MeanHR), expressed as beats per minute (bpm), was extrapolated.

**Table 3:** Name, start time and end time of the five-time windows from which the HRV parameters were extrapolated

Time window name	Start	End
Basal 1	Start of the ECG recording	Collection of the first saliva sample (T0)
Permanence	Minipig’s first entry into the kennel	Minipig’s first exit from the kennel
Basal 2	Collection of the second saliva sample (T1)	Collection of the third saliva sample (T2)
Transport	Minipig’s second entry into the kennel and transport by road	Minipig’s second exit from the kennel, after returning to the test area
Basal 3	Collection of the fourth saliva sample (T3)	End of the ECG recording

The salivary samples were analyzed using a commercial enzyme immunoassay kit (Salimetrics^®^, State College, USA) for the determination of cortisol in saliva. The internal validation of the salivary cortisol assay in pigs was conducted in the laboratory of the Department of Veterinary Sciences of the University of Pisa through assessments of repeatability and linearity.

### Statistical analysis

Data on HRV and cortisol were observed in their distribution using histograms and QQ plots, and tested for normality using the Shapiro-Wilk Test. Data was not normally distributed and was expressed as median and interquartile range (IQR). The Wilcoxon Rank-Sum Test for independent samples was used to test the differences between groups (intergroup) at different times for the parameters of interest (i.e., HRV, cortisol). The Friedman test with Nemenyi post hoc correction was used to test the differences between times (HRV: Basal 1 vs. Permanence vs. Basal 2 vs. Transport vs. Basal 3; cortisol: T0 vs. T2 vs. T3) within the group (intragroup) for the same parameters of interest. Statistical analysis was performed using RStudio (R version 4.4.2). A *p*-value ≤ 0.05 was considered significant.

## Results

**Fig. 3:**
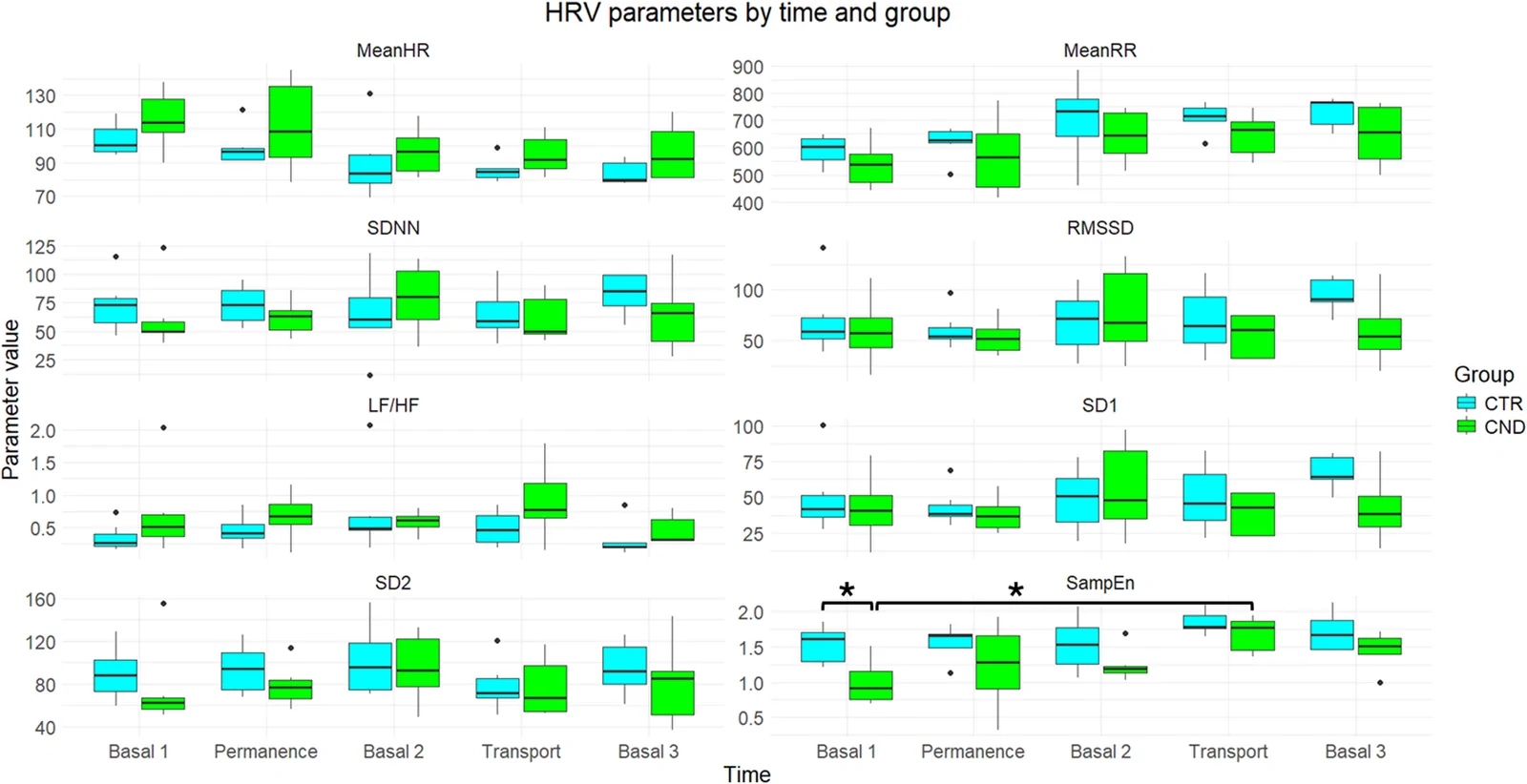
Boxplot of HRV parameters (Mean HR, MeanRR, SDNN, RMSSD, LF/HF, SD1, SD2, SampEn) by time (Basal 1, Permanence, Basal 2, Transport, Basal 3) and group (CTR and CND). In CND group, data were missing for one minipig at Transport (*n* = 12) and Basal 3 (*n* = 12). In CTR group, data were missing for one minipig at Transport (*n* = 12) and for two minipigs at Basal 3 (*n* = 11). This figure shows the outliers as black dots. Asterisks indicate statistically significant differences (*p* < 0.05)

All the minipigs in the CND group completed the conditioning training in five weeks, except for one female and one male minipig that required an additional week (i.e., six weeks total). Descriptive statistics for each HRV parameter in each of the five time windows of the ECGs are reported in Table 4 and Fig. 3.

**Table 4:** Table of HRV by time (Basal 1, Permanence, Basal 2, Transport, Basal 3) and group (CTR and CND)

	Group	Basal 1	Permanence	Basal 2	Transport	Basal 3
MeanHR (bpm)	**CTR**	100.18 (96.72-109.84)	96.68 (91.59–98.40)	83.40 (77.90-94.43)	84.80 (81.00-86.54)	79.91 (78.67–89.92)
	**CND**	113.64 (108.21-127.86)	108.81 (93.28-135.26)	96.33 (84.82-104.61)	91.80 (86.50-103.61)	92.26 (81.37-108.65)
MeanRR (ms)	**CTR**	602.60 (557.00-633.70)	628.60 (619.70-660.70)	734.30 (642.90-777.60)	714.9 (699.6-745.7)	766.30 (686.1-766.60)
	**CND**	538.00 (474.90-578.10)	566.20 (457.50-649.70)	645.60 (579.2-727.2)	664.70 (582.90-696.30)	657.90 (559.60-747.60)
SDNN (ms)	**CTR**	72.96 (57.49–79.09)	72.77 (59.47–86.06)	60.06 (53.37–79.61)	59.28 (53.59–75.96)	85.05 (72.54–99.01)
	**CND**	49.71 (49.05–58.38)	63.11 (51.02–67.82)	79.90 (60.16-102.92)	49.91 (47.68–78.23)	65.93 (41.50-74.18)
RMSSD (ms)	**CTR**	59.12 (51.50-72.55)	54.30 (51.81–63.23)	71.38 (45.96–89.19)	64.33 (47.93–93.40)	91.01 (88.62-110.06)
	**CND**	57.14 (43.17–72.69)	52.08 (40.72–61.08)	67.48 (49.78-116.45)	60.30 (33.09–74.78)	54.40 (41.79–71.47)
LF/HF	**CTR**	0.26 (0.21–0.39)	0.41 (36.67–44.76)	0.48 (0.46–0.66)	0.46 (0.27–0.68)	0.20 (0.19–0.25)
	**CND**	0.51 (0.36–0.70)	0.67 (0.54–0.85)	0.61 (0.50–0.67)	0.77 (0.64–1.19)	0.30 (0.30–0.62)
SD1 (ms)	**CTR**	41.82 (36.23–51.35)	38.44 (36.67–44.76)	50.53 (32.53–63.15)	45.55 (33.93–66.12)	64.42 (62.75–77.92)
	**CND**	40.45 (30.55–51.41)	36.85 (28.81–43.23)	47.77 (35.24–82.44)	42.68 (23.43–52.94)	38.51 (29.59–50.59)
SD2 (ms)	**CTR**	88.23 (72.65-102.19)	94.21 (74.61-108.85)	95.45 (74.19-118.14)	71.19 (66.66–84.55)	91.73 (79.97-114.69)
	**CND**	62.51 (56.54–67.13)	76.38 (65.76–83.66)	92.15 (77.34-121.67)	66.88 (54.21–96.96)	84.99 (50.70-92.02)
SampEn	**CTR**	1.61 (1.29–1.70)^A^	1.66 (1.49–1.68)	1.54 (1.26–1.77)	1.79 (1.76–1.95)	1.67 (1.47–1.88)
	**CND**	0.91 (0.76–1.16)^B, a^	1.28 (0.91–1.66)	1.19 (1.13–1.22)	1.77 (1.45–1.86)^b^	1.51 (1.39–1.63)

Legend: *bpm* = beats per minute; *ms* = milliseconds; *CTR* = control group; *CND* = conditioned group

Within the same column, different superscripts mean statistical differences over groups (intergroup): A ≠ B (*p* < 0.05). Within the same row, different superscripts mean statistical differences over time (intragroup): a ≠ b (*p* < 0.05)

**Fig. 4:**
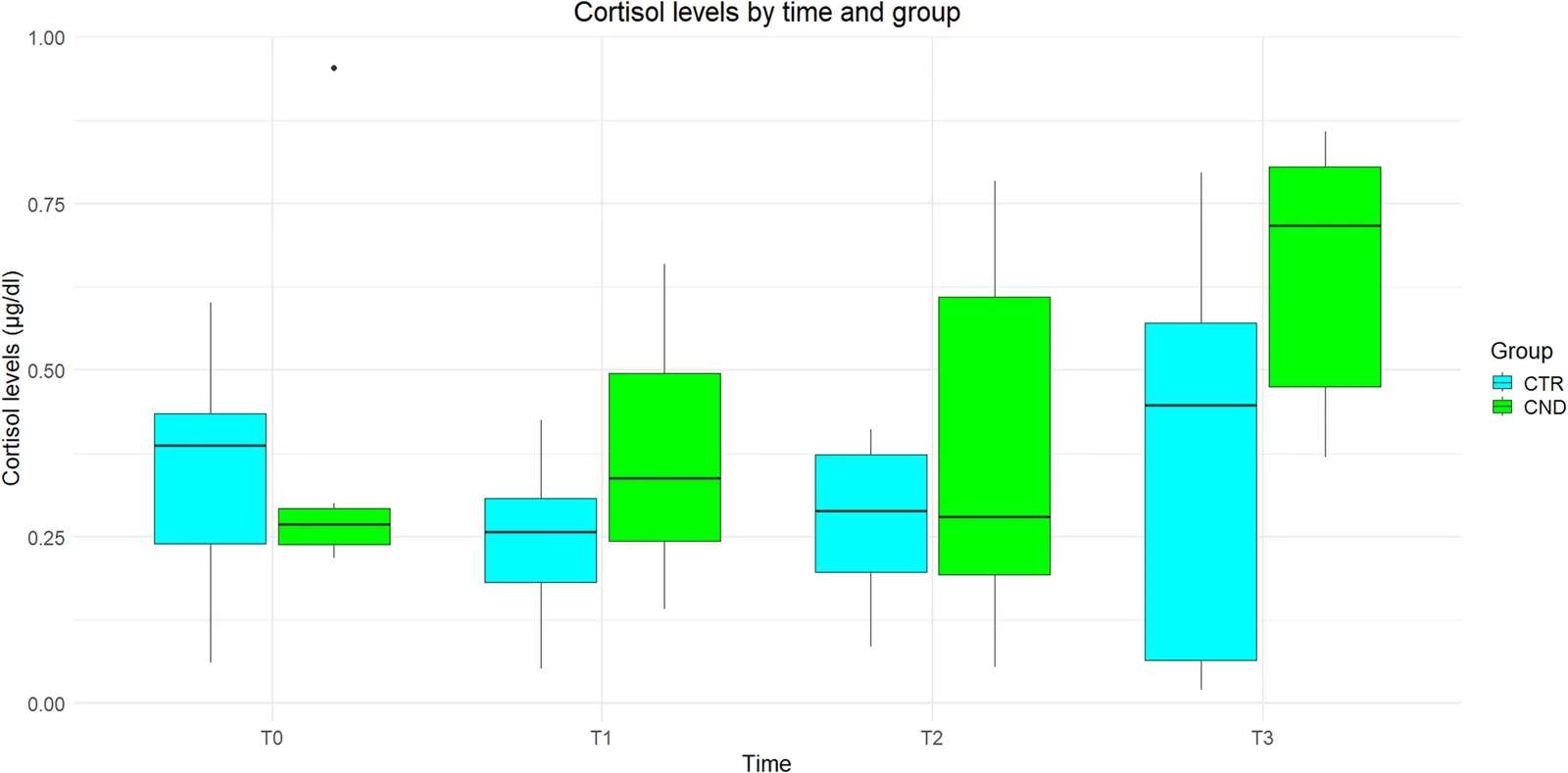
Boxplot of the cortisol concentration (µg/dl) by time (T0, T1, T2, and T3) and group (CTR and CND). No data was missing. There were no statistically significant differences

All HRV parameters were missing for one minipig in group CND at Transport and Basal 3. All HRV parameters were missing for one minipig in group CTR at Transport and for two minipigs in group CTR at Basal 3. No statistically significant differences were found in the intergroup and intragroup comparisons for HRV parameters, except for the SampEn (Table 4 and Fig. 3). For this parameter, a statistically significant difference was found in the intergroup comparison at Basal 1, with CTR minipigs showing higher SampEn values than CND minipigs (*p* < 0.05; Table 4 and Fig. 3). In addition, a statistically significant intragroup difference was found in the CND group between Basal 1 and Transport, with a higher SampEn value during Transport (*p* < 0.05; Table 4 and Fig. 3). Figure 4 shows the graphical descriptive statistics for cortisol levels. No statistical differences were found between or within groups for cortisol levels (Fig. 4).

## Discussion

This study aimed to evaluate whether a five-week kennel conditioning protocol could reduce physiological stress responses in minipigs during simulated transport. Unexpectedly, neither the CND nor the CTR animals showed clear physiological evidence of stress related to transport. These results imply that both groups may have developed resilience to the experimental procedures and transport simulation. Therefore, the extensive handling and exposure to novel, non-aversive stimuli throughout the weeks of conditioning may have substantially contributed to the animals’ adaptive responses.

Among the investigated physiological measures, only sample entropy (SampEn) showed statistically significant inter- and intra-group differences. SampEn quantifies the complexity and regularity of a time series, with higher values generally reflecting greater variability and adaptive capacity of physiological regulation (Shaffer and Ginsberg 2017). However, given the absence of consistent changes across core HRV indices, this finding should be interpreted cautiously and primarily as an exploratory indication of altered autonomic dynamics.

At baseline, CTR animals showed higher entropy values than CND animals. One possible explanation is that CND minipigs experienced a state of positive emotional arousal, potentially due to the expectation of a familiar and previously rewarded context upon entering the box, as a consequence of training (Leliveld et al. 2016; Bakhchina et al. 2018). SampEn is a general index of system complexity and is not specific to either sympathetic or parasympathetic activity. External stimuli, including emotionally relevant events, may transiently reduce complexity measures even under positive conditions (Leliveld et al. 2016). Therefore, the lower baseline SampEn observed in conditioned animals may reflect anticipatory arousal rather than reduced welfare, although this interpretation should be considered with caution. The increase in SampEn observed in CND minipigs during transport may indicate differences in autonomic regulation; nevertheless, the biological significance of this variation remains uncertain. Similar modulation of HRV dynamics following habituation or transport-related conditioning has been reported in goats and horses, where conditioning to transport or trailers has been shown to modulate HRV and reduce sympathetic dominance during road movement (Schmidt et al. 2010; Kannan et al. 2023).

No significant differences in salivary cortisol concentrations were detected between or within groups. This result diverges from what has been observed in beagle dogs, where conditioning to kenneling environments mitigated the cortisol rise typically associated with novel confinement (Rooney et al. 2007). However, our results may reflect a species-specific modulation, as adult minipigs might already possess a degree of resilience to short-term confinement and handling. It is also worth noting that in some studies, including those on goats and dogs, cortisol levels can remain elevated even in CND subjects, indicating that full adaptation might require longer exposure or different stressor types (Herbel et al. 2020; Kannan et al. 2023). Lastly, the minipigs in our study were not acclimatized to saliva sampling. Therefore, the manual saliva sampling procedures may have induced stress, cancelling out any possible differences between the groups (Fiderer et al. 2024).

Other possible explanations for the absence of differences in the physiological parameters (i.e. HRV and cortisol) of the two groups could be that neither the CTR nor the CND minipigs were accustomed to transport; the CND minipigs were only accustomed to the kennel in which they would be transported. Transport was therefore an unfamiliar stimulus for both groups. This may have cancelled out the effect of conditioning in reducing stress. An alternative interpretation is that repeated positive handling and gradual exposure to novelty throughout the experimental period enhanced behavioral flexibility in both groups. Regular human interaction, familiarization with the testing environment, and exposure to novel but non-aversive stimuli, namely the operator, experimental area, and smart textile system, may have reduced fear responses independently of the specific kennel conditioning protocol. From this perspective, the CTR group may not represent minimally handled animals typically encountered in routine transport situations. Instead, the overall management approach adopted during the study may itself have functioned as a form of implicit conditioning, contributing to the apparent absence of a marked response to stress during simulated transport. These findings support the possibility that good routine handling and positive human–animal interactions may substantially mitigate transport-related stress, eliminating the need for highly specific training procedures or reducing the required training time.

Some limitations of the study should be acknowledged. This study did not consider behavioral observations when assessing stress in minipigs. However, to achieve a multi-parameter welfare assessment, future studies should consider incorporating behavioral observations. The conditioning protocol lasted only five to six weeks and was applied exclusively to adult animals. In future studies, extending the conditioning period and comparing animals of different ages may help refine experimental protocols designed to evaluate physiological stress responses, such as HRV and cortisol measurements. Currently, the available evidence does not permit definitive recommendations on the routine kennel conditioning of research minipigs before transport. Further studies are necessary to evaluate the practical welfare benefits of this approach compared to alternative strategies, such as providing adequate acclimation periods after transport and building positive human-animal interactions. Finally, control animals underwent repeated handling and exposure to experimental procedures prior to transport simulation, which may have reduced contrasts between groups and limited the detection of conditioning-specific effects.

Additional methodological constraints should also be considered. The time taken for cortisol levels to reach the peak or to return to baseline was not recorded. This information could have revealed a possible trend in the two groups, beyond a statistical difference. Further studies should extend the cortisol sampling period until peak cortisol concentrations are reached and baseline values return to assess potential differences between the CND and CTR animal groups. The minipigs in both groups had not been acclimatized to potential confounding stimuli, such as the procedures involved in collecting saliva. Moreover, the small group sample sizes could have affected our observations and limited the statistical power. Despite these limitations, our study contributes to the growing body of literature evaluating preconditioning strategies in biomedical models. Particularly relevant is the recognition that physiological responses may not always align temporally or in magnitude (Rooney et al. 2007).

Taken together, these findings suggest that improving routine handling practices and promoting positive human–animal interactions may represent an effective and practical strategy for mitigating transport-related stress in research minipigs. Future research should aim to disentangle the relative contributions of targeted conditioning protocols and general husbandry practices, extend the conditioning and sampling period, and integrate additional physiological and behavioral indicators, including alternative biomarkers (e.g., oxytocin, catecholamines), to better characterize subtler shifts in emotional states and stress resilience.

## Conclusions

During simulated transport, five-week conditioned minipigs exhibited higher SampEn values than control animals; however, no group differences were detected in cortisol levels. The observed variation in SampEn values may indicate differences in autonomic regulation between groups; however, the exact biological significance of this finding in terms of welfare outcomes is unclear. Notably, neither group exhibited clear physiological evidence of transport-related stress. This finding suggests that repeated positive handling, familiarization with the experimental environment, and exposure to novel but non-aversive stimuli may have contributed substantially to stress resilience, potentially reducing the need for highly specific transport-conditioning protocols. These results underscore the complexity of interpreting physiological stress indicators and emphasize the necessity of multimodal approaches combining physiological, behavioral, and contextual indicators when evaluating welfare. Further studies should investigate age-related susceptibility, extend conditioning and sampling periods, and integrate additional behavioral and physiological welfare indicators to clarify the relative contributions of targeted conditioning protocols versus general husbandry and handling practices in shaping stress responses in research minipigs.

## Data Availability

The datasets generated during and/or analyzed during the current study are available from the corresponding author on reasonable request.
